# Ice Wines Under the Shadow of Global Warming: Climatic Threats, Production Mechanisms and Adaptation Strategies

**DOI:** 10.3390/foods15193422

**Published:** 2026-09-24

**Authors:** Vitaliy Kolotylo, Małgorzata Gniewosz

**Affiliations:** Department of Food Biotechnology and Microbiology, Institute of Food Sciences, Warsaw University of Life Sciences WULS-SGGW, 159C Nowoursynowska Street, 02-776 Warsaw, Poland

**Keywords:** ice wine, climate change, winter warming, wine regions, adaptation, viticulture, OIV, VQA

## Abstract

Due to its reliance on natural grape freezing at or below −7 °C, ice wine production is limited to small areas worldwide and is very susceptible to winter warming. Using CMIP6 climate projections, ETCCDI extreme temperature indices, and the most recent analytical findings from Canadian, Chinese, and European studies, this review summarises the most recent research on the effects of accelerated winter warming and its implications for ice wine production. Evidence for this warming trend spans several levels: broad climatic context is provided by Arctic warming exceeding 0.74 °C per decade; scenario-based projections indicate that Ontario’s ice wine zone may migrate northward by as far as 6° latitude by 2099; and indirect regional proxies, such as the Sum of Active Temperatures in Central Poland, show an increase of 0.07% annually since 1975 alongside more infrequent frost events. In addition to performing regional diagnostics, the review examines the physicochemical effects of freeze–thaw cycles on volatile aroma profiles, compares natural ice wine with cryoextraction alternatives through OIV and VQA governance mechanisms, and explores adaptation strategies such as varietal repositioning, geographic shifts, and precision harvest monitoring. The main research gaps are highlighted by examining factors at the vineyard level for frost threshold modelling, as well as global authenticity markers. Ice wine is certainly one of the most prominent premium agricultural products whose long-term viability depends directly on the trajectory of global climate change.

## 1. Introduction

Ice wine is a unique product from both technological and geographical perspectives. Unlike most wine styles, which benefit from warm, sunny growing seasons, ice wine production depends on prolonged periods of severe winter cold within a narrowly defined temperature range. Grapes must freeze naturally on the vine, and both harvesting and pressing must be carried out at temperatures no higher than −7 to −8 °C. Consequently, ice wine can be produced only in a limited number of regions and, even then, not every year. This dependence on highly specific climatic conditions makes ice wine a niche product, while its rarity is a key factor contributing to its premium market value [1].

Traditionally, Canada and Germany have been the leading producers of ice wine worldwide. In recent decades, however, new production regions have emerged, particularly in China, where both production volumes and consumer demand have increased substantially [2]. China has developed into an important ice wine-producing country centred on Huanren County, Liaoning Province, which holds protected geographical indication status under provincial standard DB21/T 4259—2025 [3]. Provincial and county government sources report annual ice wine production in Huanren in the range of approximately 800 to 1500 tonnes [4,5], corresponding to a stated share of over half of global ice wine output; however, this figure has not been independently verified, the two sources do not specify a common reference year, and no consolidated national statistic for ice wine production has been published by Chinese government statistical authorities. Additional ice wine production is reported in the Yalujiang Valley, Jilin Province. Canada, the leading global producer of ice wine, produces approximately 1 million litres annually. Ontario is the major ice wine-producing region in Canada, accounting for more than 90% of Canadian ice wine production, with an average annual production historically reported at approximately 850,000 L [6]. In Germany, the production of Eiswein is highly variable between vintages. Official quality-control statistics reported 3000 hL of wine in the combined Beerenauslese, Trockenbeerenauslese and Eiswein categories in 2023, while the corresponding volume was only 1000 hL in 2024 [7].

Overall, ice wine production is geographically concentrated in a limited number of countries, with China and Canada representing major production centres, while European production, particularly in Germany, is more strongly influenced by interannual climatic conditions. The available production data also indicate substantial variability between years, reflecting the dependence of ice wine production on the occurrence of suitable natural freezing conditions.

### Product Specificity and Consumer Habits

Beyond its distinctive production method, ice wine occupies a specific niche within the broader category of sweet and dessert wines. Unlike botrytised sweet wines such as Sauternes or Tokaji, whose concentration and complexity depend on the activity of *Botrytis cinerea* (noble rot) under humid autumn conditions, ice wine derives its concentration primarily from physical dehydration and natural cryoconcentration, as defined by the regulatory and technological criteria described below, rather than from microbial or enzymatic transformation of the fruit. This regulatory definition does not, however, guarantee that the berry remains intact or microbiologically inactive throughout the extended ripening period, since prolonged on-vine retention and repeated freeze–thaw exposure can themselves affect berry integrity and surface microbiota. Where these conditions are favourable, ice wine can display a comparatively clean, fruit-driven aromatic profile and a more predictable sugar–acid balance than most botrytised wines, at the cost of substantially greater production risk, since an entire vintage can be lost if a sufficiently hard and well-timed freeze fails to occur. Because yields are extremely low and the process cannot be scaled up on demand, ice wine is typically bottled in small formats, commonly 200 mL or 375 mL rather than the standard 750 mL, which reinforces its positioning as an occasion-driven luxury product rather than an everyday wine [8].

Consumer engagement with ice wine reflects this premium positioning. Owing to its intense sweetness and high price per bottle, it is typically consumed in small servings, as a dessert wine or digestif, rather than as an accompaniment to a full meal, and purchases are commonly associated with gifting, celebration, and wine tourism rather than routine consumption. Demand from Asian markets, and China in particular, has grown alongside the expansion of Chinese ice wine production, reinforcing a pattern in which rarity and prestige, rather than everyday drinkability, underpin much of the product’s commercial value [8]. Because this value depends so heavily on scarcity and authenticity, even a modest decline in the frequency of suitable freezing winters can carry economic and reputational consequences for producers.

The strong dependence of ice wine production on winter weather has become an increasingly important source of uncertainty. Global temperatures have continued to rise in recent years, with 2015–2022 comprising the eight warmest years on record [9], and both 2023 and 2024 have continued this trend among the warmest years ever measured. Winters in particular have been affected: January 2020 was the warmest January in 141 years, and February 2020 was the second-warmest February on record [10].

For ice wine producers, these climatic changes not only mean that the critical temperature threshold of −7 °C is reached less frequently, but also that winter weather has become increasingly unpredictable. This review aims to provide a comprehensive overview of current knowledge published between 2020 and 2026 concerning the relationship between global warming and ice wine production. It examines recent climate trends, grapevine physiology, production technologies, adaptation strategies, and future market prospects. In addition, it identifies key research gaps that must be addressed to safeguard this distinctive winemaking tradition.

This article is presented as a structured narrative review rather than a systematic review. To ensure systematic and up-to-date coverage of this rapidly evolving field, the literature search followed a structured, plan-driven strategy conducted between March and September 2026. A thematic outline of the review was first established, defining the specific issues to be addressed within each section and subsection; representative search concepts and terms were then formulated individually for each thematic block, including combinations such as “ice wine” or “icewine” or “Eiswein” with “climate change”, “winter warming”, “cryoextraction”, “fermentation”, “aroma”, and “authentication”, in order to retrieve the literature most relevant to that particular aspect of the review. Elsevier (ScienceDirect), Springer, and MDPI were used as publisher platforms to access full texts once relevant records had been identified, while Web of Science, a bibliographic indexing database, was used in combination with these platforms to cross check topic coverage and citation context across publishers. The AI-assisted literature discovery tool Scopus AI was used as a complementary abstract-level search aid: candidate records identified by Scopus AI as thematically relevant were then opened on the publisher platform in full text, and each record was individually read and checked against the specific claim it was intended to support before inclusion, so that AI-assisted discovery served only to locate candidate literature rather than to determine citation content. Priority was given to publications from 2020 onward, particularly those reporting new climatic data and recent developments in climate change research relevant to viticulture. Where an older publication provided a sound description of an established production procedure, analytical method, or definition that did not require verification against more recent sources, it was retained regardless of its publication date. In particular, several classical oenological reference works and dictionaries were used to provide precise, foundational definitions and technological descriptions; although these sources are of an earlier vintage, they are considered to represent a valuable and enduring element of viticultural knowledge, complementary to the recent literature used to characterise current trends and ongoing changes in the field.

## 2. The History of Ice Wine Production

The earliest evidence of ice wine production dates back to the Roman period [11]. However, these accounts most likely referred to isolated occurrences rather than the intentional, large-scale production of ice wine. Between the Roman period and the documented frost harvests of the late eighteenth and early nineteenth centuries, the surviving record contains no substantial evidence of a continuous or deliberate ice wine tradition [11,12]. This centuries-long gap most likely reflects a genuine absence of intentional production rather than a mere lack of documentation. Throughout the mediaeval and early modern periods, viticulture in the cool-climate regions later associated with ice wine, principally the German lands and neighbouring parts of Central Europe, was already oriented toward late-harvest and botrytis-affected sweet wines [13]. Winemakers in these regions therefore already possessed the practical knowledge of delayed harvesting, careful selection of overripe fruit, and slow, high-sugar fermentation needed to recognise, and eventually to reproduce deliberately, the unusual outcome of an accidental hard freeze once such an event was first recorded, as described below.

The first fully documented harvest of grapes intended for ice wine production took place in 1830, when vineyard owners in Dromersheim, near Bingen in the Rheinhessen region of Germany, were caught off guard by an unexpected frost that froze the grapes before they could be harvested [14]. However, some historical sources suggest that a similar event occurred as early as 1794 near Würzburg in the Franconia region. According to these accounts, the frozen grape clusters were initially harvested as animal feed. It was subsequently discovered that part of the juice within the berries had remained unfrozen, resulting in a highly concentrated, viscous, and exceptionally sweet liquid. The grapes were therefore pressed while still frozen, and the extracted juice was allowed to ferment, producing a wine of unexpectedly high quality.

Systematic research on ice wine and its production methods was not undertaken until the period between 1955 and the late 1960s, when pioneering studies were conducted by Dr Hans Georg Ambrosi [15]. Historical records indicate that, for many years, ice wine was produced only in very limited quantities by a small number of winemaking families and only when weather conditions were sufficiently favourable [16]. Interest in ice wine increased markedly during the 1990s, leading to a substantial expansion of production.

The regional trajectory of ice wine production diverged considerably from this point onward, reflecting differing climatic conditions, regulatory environments, and market strategies. During this period, Germany and Austria were the principal producers of ice wine, whereas Luxembourg, Croatia, Slovenia, the Czech Republic, Hungary, Romania, Switzerland, and the United States produced considerably smaller volumes [17]. Canada entered the ice wine market in the late 1980s. The continental climate of Canada’s principal grape-growing regions, characterised by warm summers and consistently cold winters, provides favourable conditions for annual ice wine production. This climatic advantage enabled Canada to become the dominant global producer in the following decades [16].

This rise was strongly reinforced by a specific reputational milestone: in 1991, Inniskillin’s 1989 Vidal ice wine won the Grand Prix d’Honneur at Vinexpo in Bordeaux [18], bringing international attention to Canadian ice wine for the first time and stimulating both export demand and further domestic investment. The subsequent establishment of the Vintners Quality Alliance (VQA) appellation and certification system in Ontario in the late 1980s (detailed in Section 4.1) [19] provided the regulatory framework that allowed this expansion to proceed under a guarantee of authenticity, further consolidating both domestic and export confidence in Canadian ice wine.

China represents the most recent wave of geographic expansion, beginning only in the 2000s as producers in Liaoning and neighbouring provinces recognised that local winter conditions could support commercial ice wine production, as discussed in detail in Section 4.4. Unlike the gradual, family-scale development that characterised Central Europe or Canada’s rapid, export-driven commercialisation, growth in China has combined suitable regional climate with substantial industry investment aimed at capturing rapidly growing domestic demand for premium and luxury wine products [4]. This divergent history illustrates a broader theme of this review: the geographic spread of ice wine production has never depended on climatic suitability alone, but also on the historical availability of technical winemaking knowledge, regulatory recognition, and market demand at the time a given region’s climate became favourable.

## 3. Global Trends in Winter Temperatures

### 3.1. Accelerated Global Warming and Record-Breaking Anomalies

On a global scale, average air temperatures have increased steadily, with the rate of warming accelerating markedly over recent decades. Analyses published by the National Oceanic and Atmospheric Administration (NOAA) and the Intergovernmental Panel on Climate Change (IPCC) indicate that the rate of increase in global surface temperature has risen from approximately 0.08 °C per decade since the late nineteenth century to around 0.18 °C per decade since 1981 [20]. This trend is particularly relevant to ice wine production because climate warming reduces both the frequency and intensity of winter freezing events, which are essential for the natural freezing of grapes on the vine [21].

Data from Phase 6 of the Coupled Model Intercomparison Project (CMIP6) indicate that Northern Europe is expected to experience a substantial decline in the frequency of cold days and severe frosts as global temperatures rise by 1.5–2.0 °C above pre-industrial levels. An increase in global warming from 0.5 °C to 2.0 °C is projected to reduce the number of cold days by 60–80%, while the cumulative cold intensity is expected to decline by 70–90%. From the perspective of ice wine production, these projections are particularly concerning, as severe frost events that currently occur approximately once every ten years may occur only once every 30–70 years in the future, with the most extreme cold events becoming increasingly rare [22].

This phenomenon is not confined to Europe. An analysis of satellite observations across the Northern Hemisphere demonstrated that more than 70% of the investigated ecoregions have experienced a statistically significant reduction in the number of days during which the land surface remains frozen. By the end of the twenty-first century, the duration of frozen ground conditions is projected to decrease by more than 30 days per year on average, with the greatest reductions expected in Northern Europe, Canada, Alaska, and eastern Russia. The authors further estimated that each 1 °C increase in mean air temperature shortens the freezing season by approximately six days, while the concurrent decline in snow cover further amplifies this effect [23].

Comparable trends have been reported across other regions of the Northern Hemisphere. On the Mongolian Plateau, winter temperatures have increased by approximately 0.41 °C per decade, accompanied by a pronounced northward shift in isotherms [24]. In East Asia, winter temperatures are increasing more rapidly than summer temperatures [25]. Similarly, in the Himalayas, winter warming has persisted and accelerated, particularly during the latter half of the observational record [26], while projections for the Tibetan Plateau indicate that winter temperatures could increase by more than 10 °C under high-emission scenarios [27].

Arctic amplification has resulted in the Arctic warming three to four times faster than the global average. Consequently, the winter warming trend accelerated from 0.22 °C per decade during 1900–2020 to 0.74 °C per decade during 1979–2020 [28].

Record-breaking temperature anomalies were also observed at Vostok Station in Antarctica in 2024 [29]. Likewise, Russia, a country historically associated with long and severe winters, recorded its second-warmest year since instrumental measurements began in 1891. In Moscow, January 2025 became only the second January on record with a mean monthly temperature above 0 °C (+0.1 °C), exceeding the long-term average by more than 6 °C [30].

Collectively, these findings demonstrate that even regions traditionally characterised by prolonged and severe winter frosts are experiencing progressively milder winters. As a consequence, the climatic conditions required for natural ice wine production are expected to become increasingly rare, posing a significant challenge to the long-term sustainability of this unique winemaking practice.

### 3.2. Mechanisms of Temperature Variability in Winter

Global warming does not occur as a linear process but is influenced by complex atmospheric dynamics that can generate localised and episodic cold extremes even under a warming climate. Understanding these mechanisms is essential for assessing the future risks associated with ice wine production.

One of the key drivers is the loss of Arctic sea ice, particularly in the Barents and Kara Seas, which is altering atmospheric circulation across the Northern Hemisphere [31]. Reduced sea ice extent affects the behaviour of the polar vortex, the jet stream, and the frequency of atmospheric blocking events. These changes modify regional winter temperature variability and may, paradoxically, contribute to episodic cold outbreaks in temperate regions despite the overall warming trend [32].

Another important mechanism involves interactions between the stratosphere and the troposphere. Sudden stratospheric warming events and fluctuations in the strength of the polar vortex can substantially influence near-surface weather conditions. Consequently, periods of anomalous warmth may alternate with unexpected outbreaks of severe cold during late winter or even early spring [33].

Volcanic eruptions also influence winter climate variability. Although volcanic aerosols generally exert a cooling effect on the global climate, major tropical eruptions may lead to warmer winter conditions across parts of the Northern Hemisphere, particularly at mid- and high latitudes, through their influence on atmospheric circulation [34,35].

These processes help explain why extreme winter cold events have not disappeared despite ongoing global warming. He et al. [36] demonstrated that rising global temperatures primarily reduce the frequency of moderate cold spells, whereas the most severe cold waves continue to occur and, in some regions, including East Asia, may even intensify as a consequence of changes in atmospheric circulation. The winter of 2020–2021 in East Asia, for example, was characterised by record-breaking or near-record-breaking minimum temperatures across northern China [37], highlighting the increasing complexity and unpredictability of winter climate under a warming world.

An important complement to these observations is the concept of the time of emergence (ToE), which describes the point at which the effects of climate change become distinguishable from natural climate variability. Schuhen et al. [38] analysed 27 indicators of extreme temperature and precipitation derived from 21 CMIP6 climate models. Their results showed that the strongest warming occurred in annual minimum temperature indices, particularly TNn (annual minimum of the daily minimum temperature) and TXn (annual minimum of the daily maximum temperature), especially across the extratropical regions of the Northern Hemisphere. By contrast, indices describing the frequency of unusually cold and warm days (TN10p, TX10p, TN90p, and TX90p) exhibited detectable climate change signals earlier than indices representing temperature extremes. In tropical regions, these changes became apparent as early as the 1910s–1930s, whereas in extratropical regions, they emerged during the 1970s–1990s [38]. These findings indicate that changes in the frequency of extreme temperature events become detectable earlier than changes in their intensity.

Changes in minimum temperatures, particularly night-time temperatures, emerge at least as early as, and in some cases earlier than, changes in daytime maximum temperatures. This distinction is especially relevant for ice wine production because grape freezing occurs primarily during nocturnal temperature minima.

Seasonal analyses performed by Schuhen et al. [38] for the winter period (December–February; DJF) confirmed the annual patterns and identified the most pronounced winter warming at high latitudes across the Northern Hemisphere.

Under the high-emissions scenario (SSP5–8.5), winter night-time minimum temperatures in these regions are projected to increase by more than 8 °C above pre-industrial levels by the end of the twenty-first century. For regions traditionally associated with ice wine production, such warming would result in winter temperatures exceeding the threshold required for the natural freezing of grapes, thereby substantially reducing the suitability of these areas for ice wine production.

From the perspective of ice wine production, the most critical climatic indicator is not the mean winter temperature but the number of days during which the minimum air temperature falls below −7 °C. Although most of the studies reviewed do not explicitly evaluate changes in this threshold, they consistently report pronounced declines in the number of frost days (daily minimum temperature < 0 °C) and in cold-temperature indices such as TN10p and TX10p (Table 1, Figure 1).

These findings indicate that the fundamental climatic requirement for ice wine production is being satisfied increasingly infrequently. Moreover, the winter season, once relatively predictable, is becoming progressively more variable and difficult to anticipate. This presents a major challenge for producers because frozen grapes must be harvested and pressed within only a few hours to preserve the unique characteristics required for authentic ice wine.

## 4. Climate Change and the Feasibility of Ice Wine Production

### 4.1. Definitions, Producing Regions, and Regulatory Frameworks

Ice wine is a naturally sweet dessert wine produced from grapes that have frozen naturally while remaining on the vine [40].

Its name reflects its unique harvesting conditions, as the grapes are intentionally left on the vine until the first sufficiently severe frost, allowing them to freeze naturally before harvest.

Ice wine is produced primarily in Austria, Germany, Canada, and China [41]. Additional producing countries include Croatia, Slovenia, the Czech Republic, Hungary, Georgia, Romania, Switzerland, and the United States [42]. Although different countries use different names for this wine, they all refer to the same product. In North America, the term icewine is used, whereas Eiswein is the official designation in Germany and Austria. In Slovenia, it is known as Ledeno vino, as Ledové víno in the Czech Republic, and as Jégbor in Hungary. Regardless of the terminology, ice wine production and labelling are subject to strict legal regulations designed to preserve product authenticity and quality (Table 2).

Two principal regulatory systems govern ice wine production, differing in several important aspects. Under the Canadian Vintners Quality Alliance (VQA) [43] system, grapes must freeze naturally on the vine, harvesting must take place at temperatures below −8 °C, and the resulting must has to contain at least 35 °Brix. Wines meeting these requirements are labelled 100% ice wine, together with the designated geographical region of origin. In addition to the ice wine category, the VQA classification includes Special Select Late Harvest (minimum 30 °Brix), Select Late Harvest (minimum 26 °Brix), and Late Harvest (minimum 22 °Brix). Canadian ice wine must contain at least 7% alcohol by volume, while the concentration of acetic acid must not exceed 2.1 g/L [43]. Furthermore, VQA-certified wines produced within designated viticultural areas (three in Ontario and four in British Columbia) must be made from recognised *Vitis vinifera* cultivars or approved hybrid varieties. At least 85% of the wine must originate from the grape variety declared on the label. Wines bearing the designation Estate Bottled must be produced exclusively from grapes grown on vineyards owned or controlled by the winery [43].

The International Organisation of Vine and Wine (OIV) [44] defines Eiswein as a wine produced exclusively through natural cryoselection, without the use of artificial freezing techniques. Grapes must be harvested and pressed at temperatures not exceeding −7 °C. The grape is required to have a minimum potential alcohol content of 15% vol. (approximately 25.3 °Brix), while the finished wine must contain more than 5.5% alcohol by volume. In addition, volatile acidity must not exceed 35 meq/L (equivalent to 2.1 g/L expressed as acetic acid), and all grapes used for production must originate from the same geographical region [44].

Both the Canadian and OIV regulatory systems are therefore designed to protect the authenticity of ice wine by prohibiting the replacement of natural freezing with artificial cryoconcentration techniques. As a consequence, ice wine production remains uniquely dependent on naturally occurring freezing conditions, making it particularly vulnerable to the effects of climate change.

Austria, another country renowned for ice wine production, has four principal wine-growing regions where Eiswein is produced: Vienna, Wachau, Styria, and Burgenland, with Wachau being the best-known production area [45]. Austrian legislation requires Eiswein to have a minimum grape sugar content of 25 °KMW (Klosterneuburger Mostwaage), a scale expressing sugar concentration as grams per 100 g of must. In Luxembourg (AOP Moselle Luxembourgeose), Vin de Glace, produced exclusively from Pinot blanc, Pinot gris or Riesling grapes frozen on the vine at ≤−7 °C, must reach a minimum natural must weight of 120 °Oechsle [46].

In Switzerland, ice wine is produced in the canton of Valais, where vineyards are located at elevations ranging from approximately 650 to 1150 m above sea level, making them among the highest vineyards in Europe. Although production volumes are very limited, Swiss ice wines are recognised for their high quality.

In Germany, ice wine is marketed under the name Eiswein [47] and belongs to the Prädikatswein classification (formerly Qualitätswein mit Prädikat, QmP), the highest quality category within the German wine classification system. Prädikatswein comprises wines produced without the addition of sugar and classified according to the natural ripeness of the grapes at harvest [47]. Eiswein represents one of the recognised Prädikat categories and is produced exclusively from naturally frozen grapes. The principal production areas are located in western Germany along the Rhine and its tributaries, particularly in the wine regions of Pfalz, Mosel, Rheingau, and Rheinhessen [40]. German law (Weingesetz) requires grapes to be frozen at harvest and pressing [48]. No specific temperature threshold is codified in German national law for this purpose, though the OIV-recommended threshold of ≤−7 °C [44] is the practical reference generally followed. Compliance with the legal requirements, including sugar and alcohol content, is verified through laboratory analyses [49].

The first Polish ice wine was produced at the Jagiellonian University Vineyard in the Małopolska Province. According to expert evaluations, the 2012 vintage exhibited an intense aroma of fresh fruit and a flavour characteristic of the Hibernal grape variety from which it was produced. The naturally frozen grapes were harvested during the night of 5–6 December 2012, when temperatures fell to approximately −12 °C. Harvesting lasted about one hour, after which the must underwent fermentation for approximately six months before the wine was matured.

By 2018, the only commercially available Polish ice wine originated from the West Pomeranian voivodeship. The first vintage was produced from 2300 kg of Johanniter grapes harvested on 6 January 2017, yielding approximately 550 L of ice wine. For the following vintage, 0.75 ha of Johanniter vineyards were reserved for ice wine production. Harvesting took place on 5 February 2018, producing 4900 kg of naturally frozen grapes. Pressing required approximately 23 h and yielded around 1000 L of wine, corresponding to approximately 2500 bottles.

At present, Polish ice wine is produced in several regions, including the West Pomeranian, Greater Poland, Lower Silesian, Lubusz, and Subcarpathian voivodeships, using grape varieties such as Souvignier Gris, Pinot Noir, Riesling, and Johanniter. Although production remains limited, these wines have gained considerable consumer interest, with individual batches frequently selling out shortly after release, reflecting the growing market potential of Polish ice wine.

Grape varieties used for ice wine differ considerably by country, reflecting both climatic adaptation and viticultural tradition. In Canada, Vidal Blanc is the dominant cultivar because of its thick skin, high frost tolerance, and resistance to fungal rot during the extended hang time required before freezing, though Riesling is also widely planted; Canada is additionally notable for producing red ice wine from Cabernet Franc and, to a lesser extent, Cabernet Sauvignon, a style rarely found elsewhere [19]. German Eiswein is produced predominantly from Riesling, valued for its high natural acidity and ageing potential, together with smaller volumes from Müller-Thurgau and other traditional white varieties [7]. In Austria, Grüner Veltliner and Welschriesling are the principal varieties used for Eiswein, alongside Riesling [45]. In China, as discussed in Section 4.4, Vidal Blanc again predominates, mirroring its use in Canada [2,42]. This varietal diversity underlies much of the sensory variation between the national ice wine styles described in Section 7.1, from the fruity, floral character typical of Vidal Blanc to the higher acidity and ageing potential associated with Riesling.

Wines therefore differ substantially from country to country and, within a country, from region to region, and these differences arise from several interacting factors rather than any single cause. Grape variety sets the base aromatic and structural profile, as outlined above. The severity and duration of the freezing event at harvest determines the degree of must concentration, with colder and more prolonged freezing generally producing more concentrated, more intensely flavoured must at the cost of a lower juice yield. Regional climate and terroir, continental in Canada and Liaoning, more maritime-influenced in the German river valleys, and high-elevation in Austria and Switzerland, shape ripening patterns and acid retention ahead of the freeze. The minimum sugar and alcohol thresholds fixed by each regulatory system (Table 2) themselves impose a floor on attainable concentration and sweetness: the markedly higher minimum must weight required under the Canadian VQA standard (35 °Brix) relative to the OIV minimum (approximately 25.3 °Brix) is consistent with the generally higher sweetness and body reported for Canadian ice wine relative to some European examples produced closer to the regulatory minimum [43]. Beyond alcohol and titratable or volatile acidity, other parameters commonly used to characterise and differentiate ice wine quality include residual sugar content, pH, glycerol concentration—which contributes to viscosity and mouthfeel (Section 6.1)— colour intensity and browning index, and, for red ice wine, total phenolic and tannin content.

**Table 2:** Comparison of principal regulatory frameworks governing ice wine.

System/Country	Freezing Criterion (Harvest & Pressing)	Minimum Must Sugar	Minimum Finished-Wine Alcohol	Other Key Requirements
Canada (VQA Ice wine)	Natural freezing on vine; harvest <−8 °C	≥35 °Brix	≥7% ABV	Acetic acid ≤ 2.1 g/L; ≥85% labelled variety; designated viticultural area required
Germany (Eiswein)	Grapes must be frozen at harvest and pressing [48]; no specific temperature is codified in German national law, though the OIV-recommended threshold of ≤−7 °C [44] is the practical reference generally followed	Same natural minimum level as required for Beerenauslese in the respective region (110–128 °Oechsle, region- and variety-dependent) [48]	≥5.5% ABV [48]	Volatile acidity ≤ 30 meq/L (~1.8 g/L as acetic acid) [50]; independent Prädikat category since 1982; grapes from a single designated region
OIV (Eiswein/Ice wine standard)	Natural cryoselection only; harvest & pressing ≤−7 °C	Min. 15% potential alcohol (~25.3 °Brix)	>5.5% ABV	Volatile acidity ≤ 35 meq/L (~2.1 g/L as acetic acid); all grapes from same geographical region
Austria (Eiswein)	Natural freezing on vine (temperature not specified in reviewed sources)	≥25 °KMW (Klosterneuburger Mostwaage)	Not specified in reviewed sources	Produced in 4 regions: Vienna, Wachau, Styria, Burgenland
Luxembourg (Vin de Glace)	Natural freezing on vine at ≤ −7 °C	≥120 °Oechsle (~28.1 °Brix)	Not specified in reviewed sources	Produced exclusively from Pinot blanc, Pinot gris or Riesling grapes
Switzerland (Valais)	Natural freezing on vine (temperature not specified in reviewed sources)	Not specified in reviewed sources	Not specified in reviewed sources	Vineyards at 650–1150 m a.s.l.; very limited production volume
Poland	Natural freezing on vine (e.g., −12 °C recorded at first Polish harvest, 2012)	Not governed by a distinct national regulatory category described in the reviewed literature	Not specified in reviewed sources	Producing voivodeships: West Pomeranian, Greater Poland, Lower Silesian, Lubusz, Subcarpathian
China [51]	Natural freezing on vine; grapes held at ≤−7 °C, harvested and pressed while frozen; no external sugar source permitted	Not fixed as a single °Brix value in the standard; classified by sweetness category (e.g., dry, semi-dry, sweet) after fermentation	≥8.0% ABV (label tolerance ± 1.0% vol)	Total acid (as tartaric acid) ≥ 4.5 g/L; classified as red or white ice wine by colour; no synthetic sweeteners, acidity regulators, colourants or preservatives permitted; heavy-metal limits per GB 2762 (e.g., Pb ≤ 0.2 mg/L)

### 4.2. The Mechanism by Which Global Warming Affects Production

Warmer winters affect ice wine production through several interconnected mechanisms. First, they delay the onset of freezing conditions, prolonging the period during which grapes remain on the vine and increasing their exposure to fungal diseases, rot, and bird damage. Although prolonged dehydration can increase the concentration of sugars and organic acids, it also substantially raises the risk of crop losses and deterioration in fruit quality [1].

Second, elevated winter temperatures can disrupt the induction and maintenance of grapevine dormancy. Under warmer climatic conditions, the duration of temperatures below 7.2 °C is reduced, limiting the accumulation of chilling units required for normal dormancy progression. Although the effects of reduced winter chilling on dormancy release are not yet fully understood, insufficient cold accumulation is known to influence budbreak, vine phenology, and vegetative development during the subsequent growing season [52].

The response to winter cold also varies considerably among grapevine cultivars. Some varieties can tolerate temperatures as low as approximately −25 °C, whereas others may suffer significant frost injury at temperatures below −17 °C. This variability complicates both frost-risk assessment and the selection of suitable cultivars under changing climatic conditions [53].

Third, increasing winter temperature variability, characterised by alternating periods of thawing and severe frost, presents an additional challenge for ice wine production. Repeated freeze–thaw cycles may enhance the development of certain aromatic compounds and contribute to greater wine complexity [54]. However, excessive or irregular freeze–thaw events can adversely affect grape quality, resulting in reduced must quality and the loss of desirable aroma compounds.

The quality parameters most directly affected by these mechanisms are the very ones that define ice wine’s regulatory and sensory identity. Delayed or insufficiently severe freezing yields less concentrated must, translating into lower must weight (°Brix, °Oechsle, or °KMW), lower final alcohol, and lower residual sugar, all of which can push a vintage below the regulatory thresholds summarised in Table 2 and, even where thresholds are still met, produce a thinner, less intensely sweet wine. Because ice wine’s characteristic aromatic complexity depends specifically on repeated freeze–thaw cycling, a reduction in the number and intensity of these cycles is expected to reduce the extent of the associated oxidative aroma transformations, for example, the conversion of linalool and geraniol into their oxidised derivatives and the accumulation of β-damascenone, furfural, and TDN, yielding a simpler, more must-like aroma profile closer to a conventional late-harvest wine than to a fully developed ice wine. Warmer, more variable pre-freeze conditions also increase the risk of fungal spoilage and uneven dehydration, which can degrade varietal aroma precursors and increase the risk of excessive or inconsistent sugar concentration at the moment of harvest, with direct consequences for fermentation performance and volatile acidity.

### 4.3. Key Production Regions—An Assessment of the Situation in Ontario, Canada

Ontario has been the world’s leading ice wine-producing region for several decades, making it an important case study for assessing the effects of climate change on the industry. More than 7284 ha of vineyards are cultivated in Ontario, with over 76% of the province’s wine grapes grown in the Niagara region [55].

Studies by Hewer and Gough [56] and Williamson et al. [57] have shown that winter minimum temperatures in Ontario have increased significantly, while the frequency of temperatures below −20 °C has declined. Although warmer growing seasons generally favour the production of table wines, they reduce the likelihood of achieving the prolonged freezing conditions required for ice wine production.

Climate model projections indicate that Ontario’s suitable ice wine production zone is likely to shift northwards by approximately 3.5° of latitude between 2021 and 2050, and by as much as 6° between 2070 and 2099 [58]. In addition to rising temperatures, vineyards in the Niagara region are expected to face increasing challenges associated with greater precipitation variability, more frequent extreme weather events, and higher pest pressure [42]. Since 1976, the mean annual temperature in the Niagara region has increased by almost 2 °C, while the frost-free period has lengthened by approximately 7.4 days. Climate projections based on the high-emissions scenario (RCP 8.5, Representative Concentration Pathway 8.5) suggest that the mean annual temperature could increase to 11.4 °C by 2050 and reach 13.2 °C by 2080. Summer warming is projected to be particularly pronounced, with mean summer temperatures increasing from 20.8 °C to approximately 25.2 °C. At the same time, the number of days with maximum temperatures exceeding 30 °C is expected to rise from the current average of 15.5 to approximately 63 days per growing season. These changes are projected to coincide with a 5–15% reduction in summer precipitation by the end of the century, substantially increasing the risk of agricultural drought [39].

In contrast, precipitation is projected to increase during the remaining seasons. Spring precipitation is expected to rise from 232.4 mm to approximately 253 mm, autumn precipitation from 228 mm to 235 mm, and winter precipitation by approximately 18% (around 37 mm), with a progressively larger proportion falling as rain rather than snow [59].

The frequency of heavy precipitation events is also projected to increase substantially, reaching approximately 2.7 times the current level, particularly during winter and spring. Frost occurrence is likewise expected to shift, with the last spring frost advancing from around 20 April to approximately 3 April, while the first autumn frost is projected to be delayed from 31 October to approximately 23 November. Consequently, the frost-free period could be extended by approximately 42 days, reaching around 231 days per year by 2080 [60].

These climatic changes are expected to substantially alter grapevine phenology. Budbreak is projected to occur up to 45 days earlier, flowering approximately 19 days earlier, and berry ripening around 37 days earlier than under historical conditions. Although an extended growing season may provide opportunities for viticulture, earlier phenological development also increases the risk of damage from late spring frosts because young shoots and developing buds become exposed during periods when frost events may still occur.

### 4.4. China—Liaoning and Yunnan

China has emerged as the fastest-growing producer of ice wine worldwide. The Huanren region in Liaoning Province has become one of the country’s principal production areas, where the Vidal Blanc cultivar has developed a strong association with the local terroir, reflecting the combined influence of environmental conditions and regional viticultural practices on grape and wine characteristics [2,54].

Consistent with global climatic trends, the winters of 2022/2023, 2023/2024, and 2024/2025 in China were characterised by a mean temperature above the 1991–2020 climatological normal, by 0.2 °C, 0.3 °C, and 0.4 °C, respectively, [61,62,63], continuing the observed trend of increasing winter temperatures.

Climate projections for Liaoning Province suggest that the production of premium ice wine will become increasingly challenging under rapid warming scenarios, despite the continued occurrence of occasional severe frost events [58]. By contrast, Yunnan Province, owing to its high elevation despite its lower latitude, may retain suitable climatic conditions for ice wine production for a longer period and could emerge as a new production region in the future.

In China, successful ice wine production depends on a narrow climatic window. Fully ripened grapes must remain on the vine for several months until they freeze naturally, and harvesting can only be carried out when air temperatures fall to approximately −10 to −12 °C. Under these conditions, the frozen grapes yield highly concentrated must with a sugar content of approximately 35 °Brix before fermentation begins. Consequently, ice wine production is exceptionally sensitive to regional climatic conditions. Throughout the extended ripening period, grapes are exposed to numerous abiotic factors, including frost, repeated freeze–thaw cycles, and dehydration, as well as biotic threats that differ in their effect on ice wine quality—noble rot (*Botrytis cinerea* under dry, favourable conditions) can concentrate sugars in a manner broadly compatible with late-harvest and ice wine styles, whereas grey rot (the same organism under wet conditions) and sour rot (associated with acetic acid bacteria and yeasts following berry splitting) degrade fruit integrity and are generally detrimental—and these risks increase if sufficiently low temperatures are delayed [64].

### 4.5. Central and Northern Europe

A detailed study conducted in central Poland (the Mazowieckie, Łódzkie, and Świętokrzyskie provinces) for the period 1975–2021 documented a systematic increase in the Sum of Active Temperatures (SAT) of approximately 0.07% per year [21]. The SAT index, defined as the cumulative daily mean temperature above 10 °C between 1 April and 31 October, is one of the principal indicators used to assess the suitability of a region for viticulture. In 2021, the average SAT in central Poland reached 2702 °C. Time-series analysis using the Bai–Perron structural breakpoint test identified a significant shift in SAT around 1997–1998. Between 1975 and 1997, SAT values were generally lower and exhibited greater interannual variability, whereas from 1998 onwards, they remained consistently higher and comparatively more stable. Based on an AR(1) autoregressive model fitted to data from 1998–2021, the average SAT for central Poland during 2022–2026 was projected to range between 2700 °C and 2760 °C. These conditions are considered favourable for the cultivation of mid- and late-ripening grapevine cultivars, including Chardonnay, which typically requires a SAT of approximately 2700–2800 °C [21].

These findings illustrate a climate paradox that is particularly relevant to ice wine production. Increasing SAT values promote grape ripening and support the continued expansion of viticulture in Poland. Cultivars such as Seyval Blanc, Solaris, Riesling, and Regent are therefore becoming increasingly suitable for commercial cultivation. At the same time, progressively milder winters reduce the frequency of temperatures below −7 to −8 °C, which are essential for the natural freezing of grapes used in ice wine production. In addition, evapotranspiration has increased across all analysed voivodeships at an average annual rate of 0.05–0.09%, indicating a growing risk of water deficits during the growing season and posing a further challenge to the production of high-quality grapes [21]. Consequently, climate change exerts contrasting effects on ice wine production: warmer summers favour sugar accumulation and fruit maturation, whereas milder winters increasingly prevent grapes from freezing naturally under the conditions required for ice wine production.

Another important consequence of climate warming is the shift in the seasonal pattern of frost occurrence. Maciejewska et al. [21] reported a decline in both the frequency and intensity of September frosts in central Poland. Although this extends the thermally active growing season and improves the ripening potential of late-maturing cultivars, it also delays the earliest possible occurrence of temperatures suitable for ice wine production, pushing harvest dates further into winter or, in some years, preventing them altogether. Comparable trends have been reported elsewhere. A systematic decline in the number of frost and ice days has been documented across East Asia [65] and for large areas of the Northern Hemisphere [23], confirming the global nature of the observed phenomenon. Similarly, long-term observations from North Korea revealed a significant decrease in the number of frost and ice days between 1960 and 2019, at an average rate of approximately 0.7 days per decade, further reducing the likelihood of natural grape freezing under suitable conditions for ice wine production [65].

## 5. Ice Wine Production Technology

### 5.1. The Traditional Production Process

Traditional ice wine production is an extremely labour-intensive, risky, and costly process. The fruit left on the vines after the standard harvest date gradually dehydrates, concentrating sugars, acids, and aromatic compounds. The key stage is when the grapes reach the freezing point; the temperature must drop below −7 to −8 °C and remain at this level long enough to allow harvesting and pressing.

In practice, producers often harvest the grapes over several periods, from mid-December to the end of January, seeking the optimal balance between must concentration and juice yield. Higher juice yields are typically achieved in December, when the berries are less dehydrated and the frost does not need to be as severe, whereas later harvests produce more concentrated must at the expense of lower yields [42]. The juice yield from pressing frozen grapes is only 15–20% of that obtained during the production of table wines, which requires vineyards intended for ice wine production to carry a significantly higher number of grape clusters, sometimes up to twice as many as vineyards producing table wines [42]. It is also necessary to protect the fruit from birds using nets.

Grapes are harvested at night or at dawn, either by hand or by machine, usually between December and January [17,49]. Because the berry pulp separates into frozen water and concentrated juice under these conditions, harvesting must take place at the correct temperature; hand-picking allows selection of the healthiest clusters, while mechanical harvesting is an efficient, less labour-intensive option for larger vineyards [40].

During pressing, most of the water remains trapped as ice together with the grape skins, while the concentrated, sugar- and acid-rich juice is extracted using basket or membrane presses [42]. Pressing takes place while the grapes are still frozen, usually outdoors and as quickly as possible before temperatures rise, with pre-chilled presses recommended to maintain low temperatures throughout [40]. In Canada, hydraulic basket presses are most common because of the high pressure and short pressing time they allow [17], whereas pneumatic presses are used less often, as their lower operating pressure makes extraction from frozen grapes more difficult [40]. The resulting yield, only 15–20% of that obtained from the same grapes pressed for table wine, is consistent with estimates reported elsewhere [66,67]. Must sugar content is monitored continuously throughout pressing, which is stopped once the juice becomes too diluted or an ice layer begins to block the press.

The settling and clarification of must are usually slow processes that can be accelerated by centrifugation. Depending on the composition of the must and the winemaker’s preferences, clarification and/or sulfidation may be carried out before fermentation. Ice wine must can, in some cases, contain sufficient yeast-assimilable nitrogen to proceed without supplementation; however, nitrogen availability depends strongly on grape variety, vineyard nutrition, degree of dehydration, yeast strain, and fermentation strategy, and supplementation with diammonium phosphate or other nitrogen sources is often still required to avoid sluggish or stuck fermentation [68]. Unlike table wines, malic acid is the main acid responsible for the acidity of both the must and the final wine, as a large proportion of tartaric acid precipitates in the berries before harvest, most likely as potassium bitartrate [69].

A yeast starter culture is then added to the must; fermentation is challenging because the extremely high sugar concentration creates osmotic pressure that inhibits yeast activity. Commercial production most commonly uses selected strains of *Saccharomyces cerevisiae* and *Saccharomyces bayanus*, the latter producing less acetic acid [70]; fermentation, typically carried out at 15–17 °C, lasts considerably longer than for table wine, often several weeks or months, [42], yielding a final product of 7–13% (*v*/*v*) alcohol and around 200 g/L residual sugar.

Spontaneous fermentation, driven by the grape’s indigenous microbiota, is a less efficient but still standard-compliant (GB/T 25504-2010) alternative that, in studies on Chinese Vidal Blanc ice wines, produced wines with a more complex aromatic profile [71,72].

One of the main challenges during fermentation is the formation of acetic acid and ethyl acetate as a consequence of the severe osmotic stress experienced by yeast cells, although the relative extent to which each compound accumulates can differ substantially, depending on the yeast strain and fermentation conditions. High osmotic stress also promotes glycerol production, which is a characteristic feature of ice wine fermentation [73].

In Canada, acidification of the must or wine by up to 4 g/L is permitted. This adjustment is generally unnecessary for Riesling ice wines but may be required for other cultivars to improve the balance between sweetness and acidity and, to a lesser extent, to maintain an appropriate pH [42].

Malolactic fermentation is generally avoided in ice wine production because it would reduce the high acidity that is characteristic of this wine style [74].

Following fermentation, the young wine is clarified and stabilised. Bentonite is commonly used to remove unstable proteins, while cold stabilisation is performed by holding the wine at temperatures a few degrees below 0 °C to promote the precipitation of potassium bitartrate. Owing to the high sugar concentration and viscosity of ice wine, this process is generally more difficult than in the production of table wines [11,66].

After stabilisation, sulphur dioxide, and in many cases potassium sorbate, are added to provide antioxidant and antimicrobial protection and to prevent unwanted microbial activity, including malolactic fermentation [74]. Finally, because of the high residual sugar content, the wine is sterile-filtered before bottling [11,42,66].

### 5.2. Problems That Arise During Fermentation

Fermentation of ice wine must is more challenging than conventional table wine fermentation and typically much longer [75], because the high sugar concentration exposes yeast to severe hyperosmotic stress, which drives the formation of acetic acid as a stress by-product [76] and causes metabolic changes, reduced growth, slower fermentation and, in some cases, undesirable sensory characteristics [77].

Fermentation may continue for months without always reaching the desired ethanol concentration, since hyperosmotic stress in both *Saccharomyces cerevisiae* and *Saccharomyces bayanus* causes cell shrinkage and reduced biomass accumulation [78], increased glycerol production [79], and increased volatile acidity [80].

At elevated concentrations, the resulting acetic acid imparts an undesirable vinegar-like aroma; approximate reported concentration ranges for ice wine, as well as their relation to the wine sensory threshold for volatile acidity, are further considered together with the wider physicochemical picture in Section 6.1.

These fermentation difficulties are not independent of the climatic pressures discussed in Section 4.2. As warmer, more variable winters increasingly force producers to either harvest at lower, less concentrated sugar levels in order to catch a shorter freezing window, or delay their harvest until they experience more erratic conditions that can produce unexpectedly high sugar spikes once a hard freeze finally occurs, the severity of the osmotic stress described above, and consequently the risk of sluggish fermentation and excessive volatile acidity, is becoming less predictable from one vintage to the next.

### 5.3. Artificial Freezing—Technology vs. Authenticity

In response to increasing climatic challenges, alternative technologies such as artificial grape freezing in cryogenic chambers and fractional cryoconcentration have been developed (Table 3). The resulting products, sometimes referred to as “cryowine”, can reproduce some of the sensory characteristics of natural ice wine. However, wine regulations in Canada and Germany, as well as OIV standards, explicitly prohibit their marketing under the names Icewine or Eiswein.

This distinction is important not only from a legal perspective but also because the natural freezing of grapes on the vine produces a unique chemical and aromatic profile that cannot be fully replicated under controlled conditions. This is mainly due to the complex freeze–thaw cycles and the influence of microorganisms naturally present on the grape surface.

Cryoextraction is an alternative method of concentrating grape must. The grapes are frozen at temperatures between −5 °C and −10 °C, before being crushed and pressed [81]. This technique is commonly used in warmer wine-producing regions, including California, Australia, New Zealand, Spain, and Argentina, where natural freezing conditions rarely occur. Producers using this method argue that the way in which the grapes are frozen has little influence on the final product and that artificial freezing offers additional advantages, including protection against microbial spoilage and pest damage [40]. Cryoextraction also enables producers to determine the desired juice concentration earlier and eliminates the need to leave grapes on the vine for several months, thereby avoiding the risks associated with prolonged field exposure and winter harvesting [66].

The process differs depending on the harvesting method. For manual harvesting, the grapes are placed in small containers and transferred to a freezing chamber. After freezing, they are manually loaded into the press. In contrast, grapes harvested mechanically are first dried and then frozen in bulk using industrial freezing equipment. Liquid CO_2_ or N_2_ is commonly used as the cooling medium [82,83]. Nevertheless, producers of traditional ice wine maintain that natural freezing is essential for achieving the characteristic quality of authentic ice wine. They argue that prolonged ripening on the vine under natural winter conditions allows the grapes to develop the full aromatic complexity that cannot be reproduced by artificial freezing [2].

The principle of cryoextraction is based on the formation of ice crystals within the berries during freezing. These crystals rupture the pectin–cellulose structure of the skin cells, disrupting the grape tissue and facilitating the release of compounds from the skins into the must. The freezing rate is a key technological parameter because it directly affects the extent of tissue disruption and, consequently, the extraction efficiency and final wine composition [84]. Two related technological methods are also important here: cryoextraction proper (pressing partially thawed berries) and supraextraction (thawing before pressing)—both produce wines that are fresher, better-structured, and have a longer finish than wines made using traditional maceration.

Detailed comparative studies of two cryogenic techniques applied to the Muscat of Alexandria cultivar, ultra-rapid mechanical freezing (UF, to −28 °C within 15 min) and liquid nitrogen freezing (LN: immersion in liquid nitrogen, which boils at approximately −196 °C, for less than 1 min), revealed clear differences in the chemical composition and sensory profile of the resulting wines [84]. It should be noted that this trial compared only industrially frozen wines (UF, LN) against an unfrozen reference must, without any naturally frozen ice wine sample. It documents the post-harvest effects of cryoextraction on wine composition, rather than providing direct evidence on the equivalence, or difference, between artificial and natural ice wine.

Both cryogenic treatments increased the total polyphenol content compared with the control wine (138 mg/L gallic acid equivalents in the control versus 172 and 167 mg/L for the LN and UF treatments, respectively). This increase was attributed to greater disruption of the grape skins, which facilitated the extraction of phenolic compounds.

The greatest differences were observed in the terpenoid profile. In wines produced using liquid nitrogen freezing, the concentrations of all analysed terpenes exceeded 150% of those in the control wine, with β-myrcene reaching nearly 600%, nerol 301%, geraniol 332%, and eugenol 310% of the control values. In contrast, among the UF wines, only β-myrcene was present at a markedly higher concentration than in the control. The concentration of 1-hexanol, a compound responsible for herbaceous notes, increased by 250% in LN wines and by 50% in UF wines, most likely as a result of enzymatic oxidation of fatty acids following disruption of the grape skin cells [84].

Among the aldehydes, nonanal showed the greatest increase, reaching 482% of the concentration measured in the control wine. This compound contributes citrus-like aroma notes. Ethyl esters, which are responsible for the fruity and floral character of young wines, also increased substantially in the LN treatment. The concentrations of isoamyl acetate, ethyl butyrate, and ethyl decanoate reached 858%, 446%, and 374% of the control values, respectively.

Sensory evaluation performed by a panel of 12 trained assessors confirmed the chemical analyses. The control wines received the lowest scores for aroma intensity, whereas both LN and UF wines were rated significantly higher for fruity and floral attributes. In addition, the assessors were able to distinguish LN wines from UF wines based on their more pronounced menthol-like notes, which were associated with higher concentrations of β-myrcene and terpineol. Overall, wines produced using liquid nitrogen freezing received the highest sensory scores.

Direct evidence bearing on the question of natural-versus-artificial ice wine equivalence comes from a study on cv. Vidal, which compared wines produced from naturally frozen, refrigerator-frozen and rotary evaporation-concentrated high-sugar musts, all vinified under identical fermentation conditions. Significant differences were found in the levels of most individual phenolic acids and flavan-3-ols among the three juice types, and these differences persisted in the finished wines regardless of the yeast strain used. Linear discriminant analysis based on vanillic acid, caffeic acid, epigallocatechin gallate, gentisic acid and syringic acid content clearly separated the naturally frozen (authentic ice wine) samples from both the refrigerator-frozen and the concentrated wines; the absence of detectable caffeic acid together with a higher epigallocatechin gallate content were identified as characteristic markers of authentic ice wine, whereas concentrated wines showed markedly elevated gentisic and caffeic acid but lower vanillic and syringic acid levels. This study therefore provides direct chemical evidence that naturally frozen ice wine possesses a distinct phenolic fingerprint that is not reproduced by either refrigeration or must concentration [85].

These findings demonstrate that cryogenic technologies can substantially enhance the aromatic profile of wines. However, as the direct chemical comparison above shows, they cannot reproduce the unique combination of biochemical processes that occurs when grapes freeze naturally on the vine over several weeks. This remains the principal difference between cryowine and authentic ice wine [85].

## 6. Chemical Composition and Aromatic Profile of Ice Wines

### 6.1. Physicochemical Characteristics

Ice wine is characterised by an exceptionally high concentration of sugars (35–42 °Brix in the must), organic acids, and aromatic compounds, resulting from both the natural freezing process and the delayed harvest. The high sugar content, together with the glucose-to-fructose ratio, which can influence fermentation behaviour, and the naturally high acidity, create the characteristic balance between intense sweetness and refreshing acidity that defines this wine style. The exceptionally high sugar concentration can, however, strongly affect fermentation performance [86]. In a study of Vidal ice wine, increasing the initial sugar concentration from 370 to 450, 500 and 550 g/L progressively reduced yeast viability, sugar and YAN consumption, and ethanol production, with final ethanol concentrations of 130.26, 117.83, 106.14 and 90.85 g/L, respectively. At initial sugar concentrations of 500 and 550 g/L, ethanol production decreased to 106.14 and 90.85 g/L, respectively, while acetic acid increased to 2.14 and 2.86 g/L, indicating that excessive sugar concentrations can impair fermentation efficiency and alter metabolite production. In contrast, the 370 and 450 g/L treatments produced wines described as clear, pleasant-tasting, well balanced, and having a superior aftertaste. The glucose-to-fructose ratio also appears to be relevant to effective ice wine fermentation, with a ratio between 1:1 and 1.7:1 reported as preferable [76].

The high osmotic pressure of ice wine must promotes glycerol production by yeast, contributing to the wine’s full-bodied texture and viscous mouthfeel. Although acetic acid concentrations naturally increase during the fermentation of high-sugar musts, producers generally aim to keep them within the regulatory limit of 2.1 g/L; however, this limit is not always met, and outcomes depend strongly on fermentation conditions and yeast management. Furthermore, spontaneous fermentation generally produces lower concentrations of acetic acid than fermentation with commercial yeast strains [2].

### 6.2. Aromatic Compounds and Their Formation

The aromatic profile of ice wine results from the interaction of several factors, including the varietal characteristics of the grapes, biochemical changes during freezing and freeze–thaw cycles, the activity of yeasts and bacteria during fermentation, and transformations that occur during ageing. Low temperatures in the vineyard activate secondary metabolic pathways in the berries, leading to increased accumulation of aromatic compounds even before harvest [54].

Vidal Blanc must is dominated by higher alcohols, C13-norisoprenoids, and terpenes [87]. Following fermentation, the volatile profile shifts towards esters, higher alcohols, and organic acids. Compounds derived from phenylalanine, such as benzyl alcohol and 2-phenylethanol, contribute honey, floral, and rose aromas, whereas ethyl esters, including ethyl caprylate, ethyl laurate, and ethyl phenylacetate, are responsible for fruity, sweet, and honey notes. C6 and C9 alcohols, which initially impart green and grassy characteristics to the must, are further transformed during fermentation [2,88].

Spontaneous fermentation promotes a more pronounced development of the characteristic ice wine aroma, enhancing honey, dried fruit, floral, and subtle nutty notes while reducing the green and grassy aromas typical of the must. This effect is attributed to the activity of a mixed community of yeasts and bacteria rather than *Saccharomyces cerevisiae* alone [89]. Odour activity values (OAVs) indicate that several esters and aromatic alcohols exceed their sensory thresholds in Chinese Vidal Blanc ice wines, identifying them as plausible contributors to the aroma profile; however, an OAV above 1 marks a compound only as a candidate odorant, since actual sensory contribution also depends on matrix effects, masking, suppression, and synergistic interactions among odorants [90].

Establishing which detected compounds actually drive a given aroma attribute therefore requires a stronger evidentiary chain than OAV screening alone: GC–MS detection and quantification, followed by GC–O and/or OAV screening, followed by statistical association with sensory descriptors, and finally aroma reconstitution or omission testing to confirm a causal sensory contribution. Applying this framework to ice wine, partial least squares regression on 79 quantified volatiles across 18 commercial ice wines identified several higher alcohols, γ-lactones, fatty acid ethyl esters, monoterpenes, furaneol, and homofuraneol as positively associated with the “honey” attribute, while sotolon, lactones, β-damascenone, and oak-derived volatiles were associated with “caramel” and “dried fruit”. Subsequent aroma reconstitution and omission experiments confirmed these statistical associations at the sensory level: omitting monoterpenes produced the largest decrease in “honey” and “floral” intensity together with an increase in “caramel” character; omitting lactones reduced “caramel” and “coconut” notes; and omitting furanones (sotolon, furaneol, homofuraneol) reduced both “caramel” and “honey” intensity. Reconstitution further showed that terpenes alone enhanced “honey”, “floral”, and “peach/apricot” notes but suppressed “caramel”, whereas lactones and furanones acted additively or synergistically to enhance “caramel” and “dried fruit”; no single compound reproduced any of these three characteristic aromas in isolation. These results show that exceeding an odour threshold identifies only a candidate odorant, whereas reconstitution and omission testing are required to establish an actual, and often interactive, sensory contribution [90].

In contrast to spontaneous fermentation, controlled co- and sequential fermentation with selected non-*Saccharomyces* yeasts provides an alternative oenological strategy for modulating the aromatic profile of ice wine. In Vidal Blanc ice wine, mixed fermentation of *Metschnikowia pulcherrima* with *Saccharomyces cerevisiae* resulted in higher contents of acetate esters and β-damascenone and lower C6 alcohols compared with *S. cerevisiae* monoculture. Fermentation with *Hanseniaspora vineae* and S. cerevisiae produced particularly high levels of higher alcohols, including isobutanol, isopentanol, and 2-phenylethanol, as well as acetate esters such as 2-phenethyl acetate and isoamyl acetate, together with cis-rose oxide, β-damascenone, and phenylacetaldehyde. Sequential inoculation further increased aroma diversity and resulted in higher intensities of fruity, floral, and sweet attributes compared with simultaneous inoculation [91]. These findings indicate that controlled non-*Saccharomyces* co- and sequential fermentation can be used to selectively modify the formation of volatile compounds and provide an additional oenological strategy for tailoring the aromatic complexity of ice wine.

Consistent with this, a pure-fermentation screening of 11 indigenous non-*Saccharomyces* strains against a commercial *S. cerevisiae* reference in Vidal ice wine found that *Saccharomycopsis crataegensis* and *Hanseniaspora uvarum* most effectively enhanced sweet and floral sensory attributes, coinciding with elevated ethyl butanoate, ethyl isobutyrate, and phenethyl acetate and with strain-unique volatiles, including nerolidol, δ-decalactone, and γ-decalactone, that were not detected in the reference strain. By contrast, the strain that produced the largest overall amount of acetate esters (*Pichia kluyveri*) also produced the most pronounced nail-polish and alcohol off-notes, indicating that the aroma benefit of non-*Saccharomyces* fermentation is strain- and compound-specific rather than uniformly positive [72].

Freeze–thaw cycles also influence aroma development. Bowen and Reynolds [92] demonstrated that the concentrations of linalool and geraniol decrease over time, whereas the levels of their oxidised derivatives, including linalool oxides and nerol oxides, increase. Changes were also observed in the concentrations of β-damascenone, furfural, and TDN, reflecting oxidation processes associated with fluctuating winter conditions.

Taken together, the compounds discussed above fall into a small number of chemical classes whose relative balance defines the characteristic ice wine bouquet: higher alcohols and C13-norisoprenoids inherited from the must, fatty acid ethyl esters, and acetate esters formed during fermentation, monoterpenes and their oxidised derivatives generated by freeze–thaw cycling, and the furanones and lactones (sotolon, furaneol, homofuraneol, and the γ- and δ-lactones) responsible for the characteristic honey, caramel, and dried-fruit notes [90]. One particular norisoprenoid, TDN (1,1,6-trimethyl-1,2-dihydronaphthalene), accumulates during the extended hang time and subsequent ageing typical of Riesling-based ice wine and is widely considered principally responsible for the petrol- or kerosene-like note that distinguishes aged German Riesling Eiswein from the more purely fruity, floral profile of Vidal Blanc ice wine, consistent with the regional sensory differences [93].

## 7. Sensory Characteristics of Ice Wines

### 7.1. Typical Profile and Changes over Time

Young ice wines are characterised by intense fruity and floral aromas. In the case of Vidal Blanc, these are typically described as apple, citrus, floral, honey, and peach notes, accompanied by pronounced sweetness and refreshing acidity. During ageing, the fresh fruity aromas gradually diminish, giving way to more complex, slightly oxidative notes, including nutty, caramel, and raisin aromas [92]. Ice wines have an ageing potential rarely found among sweet wines, as their high acidity acts as a natural preservative, allowing them to evolve over many years.

Beyond aroma, appearance and mouthfeel contribute substantially to the overall sensory impression. Young ice wines typically show pale golden to amber hues that deepen toward copper and amber-brown tones with age, alongside the honey- and caramel-like oxidative notes described above, while their high glycerol content gives them a viscous, full-bodied texture on the palate that is unusual among white wines and is often described by tasters as an oily or syrupy mouthfeel [41,92].

The sensory characteristics of ice wines are influenced by the yeast strain, region of origin, and vintage. Research by Cliff et al. [41] showed that ice wines from Ontario are characterised by the greatest intensity of fruity and floral aromas and a distinctive golden-copper colour. In contrast, wines from British Columbia received the highest scores for sweetness and length of finish, whereas German wines were distinguished by pronounced nutty and oily notes together with the highest perceived acidity.

### 7.2. Sensory Consequences of Yeast Strain, Vineyard Management, and Harvest Practices

Erasmus et al. [77] found that wines fermented with the *S. cerevisiae* strains N96 and EC1118 were of the highest quality, exhibiting a light yellow colour and low levels of sulphur-related aromas. Kontkanen et al. [68] demonstrated that, even when the same yeast strain (*S. cerevisiae* K1-1116) was used, the inoculation method significantly influenced the sensory profile. Direct inoculation without micronutrient supplementation produced wines with raisin and spicy notes, whereas the addition of micronutrients enhanced honey and orange aromas. Gradual yeast acclimatisation promoted the development of peach and terpene aromas.

According to Bowen and Reynolds [92], harvest date and yield are among the main factors influencing the concentration of volatile aroma compounds in Vidal Blanc and Riesling ice wines. Harvesting too early results in wines with lower aromatic complexity, whereas delayed harvesting produces more concentrated wines but increases the risk of yield losses. Lower yields are also associated with higher concentrations of monoterpenes and a more pronounced ripe fruit character, similar to that observed in table wines.

## 8. Production Economics and Market Trends

Ice wine production is among the most expensive forms of winemaking. The extremely low pressing yield (15–20% of that obtained in table wine production), the need to protect grapes from birds, climate-related risks, and the necessity of monitoring weather conditions over several months all contribute to production costs that are substantially higher than those of table wines. In many seasons, producers face a difficult decision: wait for sufficiently low temperatures or risk losing the entire crop [11].

Global ice wine production is estimated to exceed 1000 metric tonnes annually, with Canada (primarily Ontario) accounting for approximately 80% of total production, Germany contributing an additional 10–15%, and China rapidly increasing its share of the market [4,6,7].

The high price of ice wine, resulting from both its costly production and limited availability, firmly places it within the premium wine segment. While this helps protect producers from price competition, it also limits consumer accessibility and increases the risk of product counterfeiting.

The economic paradox of ice wine production is that climate change may, in the short term, increase both the prestige and market value of these wines as they become increasingly rare. In the long term, however, the same climatic changes threaten the environmental conditions required for their production. At the same time, growing interest in ice wines across Asia, particularly in China and Japan, is creating new market opportunities while highlighting the need for greater consumer awareness and effective protection of product authenticity.

It is also worth noting the growing importance of ice cider, the apple counterpart of ice wine, as well as similar products made from other fruits. These beverages can be produced under climatic conditions that are more readily achieved than those required for ice wine production and therefore represent an attractive market niche. Nevertheless, they remain largely outside the scope of current scientific research [94]. Ice cider originated in the Canadian province of Quebec and is characterised by intense sweetness and a concentrated apple flavour resulting from production methods such as cryoextraction [95].

## 9. Authenticity, Forgeries, and Analytical Methods

The high price and prestige of ice wines make them an attractive target for fraud and adulteration [96]. The most common forms include the sale of cryoconcentrated products (cryowine) as ice wine, the addition of glucose–fructose syrup or concentrated grape juice, and the use of flavourings. Detecting such adulteration requires advanced analytical techniques.

Several complementary analytical approaches may be considered for the authentication of ice wine, although they currently differ substantially in how directly they have been validated for this specific matrix. Stable isotope analysis based on the ^13^C ratio has been successfully applied to distinguish sugars from grape-derived and non-grape sources in wine and other products [97]. However, its application specifically to ice wine authentication remains insufficiently investigated. In contrast, Hermann [98] demonstrated the potential of δ^18^O measurements for ice wine authentication by exploiting the isotopic differences associated with cryoconcentration. Other physicochemical ratios, such as the malic acid-to-sugar ratio, have been proposed as potential markers of natural freezing, since malic acid degrades progressively during prolonged on-vine exposure while sugar continues to concentrate; however, this relationship has not yet been systematically validated as an authentication marker specifically for ice wine and should be regarded as a prospective rather than an established approach. GC–MS fingerprinting of volatile organic compounds, combined with electronic nose (E-nose) analysis and multivariate statistical methods such as partial least squares discriminant analysis (PLS-DA), can provide comprehensive characterisation and discrimination of food products based on their volatile profiles, including differentiation according to geographical origin [99]. This capability, however, has not yet been demonstrated specifically for ice wine authentication. Near-infrared (NIR) and mid-infrared (MIR) spectroscopy likewise offer rapid, non-destructive screening potential for wine authentication in general, but their application to ice wine authentication specifically remains to be demonstrated. Overall, the development of validated, universally accepted authenticity markers for ice wines remains an important challenge requiring further interdisciplinary research [85].

## 10. Adaptive Strategies for Ice Wine Production

### 10.1. Plant Material and Vineyard Management

The selection of grapevine cultivars, clones, and rootstocks is the first line of defence against climate change. Cultivars with a later ripening period allow grapes to remain on the vine for longer without becoming overripe during warmer autumns. Vidal Blanc, owing to its thick skin and high frost tolerance, is among the cultivars best suited to ice wine production in cold-climate regions such as Canada; suitability nonetheless depends on the specific region and its dominant climatic risks [100]. Vidal Blanc’s suitability is well established in Canada, where unlike most inter-specific hybrids, it is explicitly recognised as an eligible variety for ice wine under Ontario’s VQA regulation [43], and in China, where no cultivar-based restriction applies under GB/T 25504-2010 [51]. In the EU Prädikatswein/Qualitätswein framework governing German and Austrian Eiswein [44,45], however, eligible varieties are generally restricted to *Vitis vinifera*, so Vidal Blanc’s applicability there is legally, and not only climatically, limited. However, the development and evaluation of new cultivars and clones adapted to changing climatic conditions remain important areas for future research [101,102].

Viticultural practices, including pruning, canopy management, and irrigation, must be adapted to increasingly earlier grapevine phenology. The use of drought-tolerant cultivars together with soils that have greater water-holding capacity can improve vineyard resilience to irregular rainfall and prolonged periods of water deficit [103,104].

### 10.2. Geographical Shifts in Production Zones

Climate model projections indicate that the suitable ice wine production zone in Ontario will shift northwards by approximately 3.5° between 2021 and 2050 and by 6° between 2070 and 2099, while in Yunnan, the potential production area may extend northwards to 28.5° N [39,58]. The selection of new vineyard sites should take into account factors such as higher elevation, favourable slope aspect, and adequate soil depth [105,106].

However, relocating ice wine production to cooler regions is a long-term process that requires substantial financial investment and the gradual establishment of a new terroir.

### 10.3. Risk Monitoring and Management

In the face of increasing climate variability, the precise monitoring of local weather conditions is becoming increasingly important. Weather stations installed in vineyards, early warning systems, and high-resolution winter weather forecasting models can help optimise harvest timing and minimise crop losses [107,108]. Flexible harvest planning across several potential harvest dates, together with the ability to rapidly mobilise harvesting teams, will remain key factors for successful ice wine production in the coming decades [109].

## 11. Future Directions

Despite growing interest in ice wine as a wine style highly vulnerable to climate change, several fundamental issues remain insufficiently studied.

There is a lack of precise, long-term data on changes in the number of days with temperatures below −7 °C in the main ice wine-producing regions. Most available climate studies use a threshold of 0 °C or rely on statistical indices that do not directly reflect the technological requirements for ice wine production [59,110].

The relationship between winter warming, cold accumulation, and the release of grapevines from dormancy remains insufficiently understood, despite its direct influence on grapevine phenology and harvest timing [52]. There is also a lack of comprehensive studies linking harvest date and yield to the chemical composition and sensory profile of the final ice wine. Although Bowen and Reynolds [92] carried out pioneering work in this area, and research groups in China are publishing an increasing number of studies on Vidal Blanc [54,76,87,100], many aspects of this relationship remain unresolved.

In wine microbiology, there is still a need for comprehensive studies investigating both the composition of microbial communities present on grapes and in must, as well as the changes in volatile organic compounds (VOCs) that occur during fermentation and wine ageing. Such studies, combining metagenomic approaches with HS-SPME-GC-MS analysis, could improve our understanding of the relationship between grape-associated microflora and aroma development in ice wines [2,87].

A better understanding of the role of indigenous microorganisms under conditions of low temperature and high osmotic pressure is essential for preserving the distinctive characteristics of ice wines associated with their local terroir [111].

The market for ice cider and similar products made from fruits other than grapes remains largely unexplored, despite growing commercial interest in these beverages. Likewise, there is still a lack of globally validated authenticity markers for ice wines, which are essential for effective protection against fraud and adulteration [94].

Addressing these research gaps requires an interdisciplinary approach combining climatology, plant physiology, oenology, sensory science, and food science, while also considering economic and market perspectives. It is equally important to establish continuous, high-resolution temperature datasets covering the period 2000–2026, with particular emphasis on local winter extremes at the vineyard level [30].

## 12. Conclusions

Climate data from recent decades clearly demonstrate that global warming is no longer an abstract future threat but a process already affecting ice wine production worldwide. Winter temperatures are increasing particularly rapidly in polar and high-latitude regions, which have traditionally been associated with severe winters.

For ice wine production, this means that the key technological requirement—a temperature below −7 to −8 °C—is being met less frequently, while winter conditions are becoming increasingly unpredictable. Climate models project a gradual shift in suitable production areas towards higher latitudes and elevations; however, this transition will require active adaptation by producers, investors, and policymakers rather than occurring naturally.

At the same time, ice wine retains its unique status as a product with a distinctive chemical and sensory profile resulting from natural processes that cannot be fully reproduced using current technologies. This authenticity represents both its greatest market strength and its greatest vulnerability in the face of ongoing climate change.

This authenticity also underpins the consumer demand: because rarity, provenance, and a sensory profile shaped by natural freeze–thaw cycling cannot be substituted by artificial concentration without yielding a different, lower-value product, the same climatic pressures that threaten production volumes also threaten the specific aromatic complexity, from freeze–thaw-driven terpene oxidation to the honey, caramel, and dried-fruit notes, on which that value depends.

Adaptation strategies, including the selection of suitable cultivars, flexible harvest management, and the relocation of vineyards to more favourable regions, may help sustain ice wine production but cannot eliminate the risks associated with a warming climate. The future of ice wine will depend not only on producers’ ability to adapt but also on continued scientific research to address existing knowledge gaps and support evidence-based strategies for preserving this unique winemaking tradition.

## Figures and Tables

**Figure 1:**
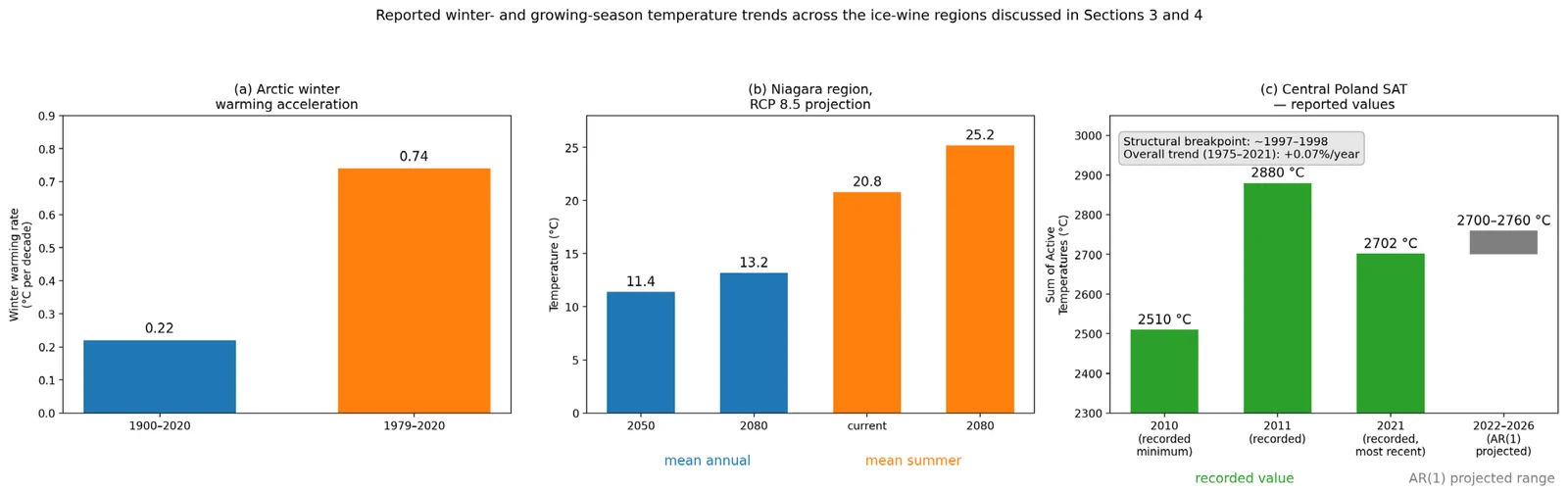
Reported winter- and growing-season temperature trends across the ice wine regions discussed in Section 3 and Section 4. (**a**) Acceleration of the Arctic winter warming rate in 1900–2020 and 1979–2020 [28]; (**b**) reported RCP 8.5 projections of mean annual and mean summer temperature for the Niagara (Ontario) region [39]; (**c**) reported summary statistics for the Sum of Active Temperatures (SAT) in central Poland, 1975–2021, shown as discrete values rather than a continuous series: the observed long-term linear trend (+0.07%/year), the identified structural breakpoint (~1997–1998), the reported 2021 endpoint value, and the projected 2022–2026 range [21].

**Table 1:** Hierarchy of climatic evidence relevant to ice wine feasibility, from direct thresholds to indirect proxies and scenario-based projections.

Evidence Tier	Indicator/Metric	Representative Reported Finding
1. Direct ice wine threshold	Harvest/press temperature ≤−7 to −8 °C	Regulatory threshold itself (VQA, OIV, Chinese Standard GB/T); most reviewed studies do not directly evaluate trends in this specific threshold
2. Indirect climatic proxies	Frost days, TN10p/TX10p, SAT, evapotranspiration, frozen-day duration, night-time minima (TNn/TNx)	Frost days and cold-percentile indices declining broadly; central-Poland SAT +0.07%/yr (1975–2021); >70% of N. Hemisphere ecoregions show significant reduction in frozen-surface days
3. Scenario-based projections	CMIP6/SSP1-2.6/SSP5-8.5 climate model projections	Ontario ice wine zone projected to migrate north 3.5° (2021–2050) to 6° (2070–2099) latitude; cold-day frequency projected to fall 60–80% between 0.5 and 2.0 °C warming levels; winter night minima in high latitudes projected +8 °C under SSP5-8.5 by 2100

**Table 3:** Comparison of natural and artificial grape-freezing/concentration technologies used to produce ice wine or ice wine-like products.

Method	Freezing Conditions	Typical Use/Region	Ice wine Label Permitted?	Key Characteristics
Natural ice wine (on-vine freezing)	Natural exposure on the vine for weeks; harvest/press ≤−7 to −8 °C	Canada, Germany, Austria, China, Poland, etc.	Yes	Juice yield only 15–20% of table wine; unique aroma from prolonged freeze–thaw cycles and natural microbiota
Cryoextraction	Post-harvest freezing of harvested berries, typically at −5 °C to −10 °C in chambers before pressing of partially thawed berries; experimental studies have additionally tested faster freezing rates within this category, e.g., ultra-rapid mechanical freezing (−28 °C within 15 min) and liquid nitrogen freezing (approximately −196 °C, the boiling point of liquid nitrogen, in <1 min)	Warmer regions: California, Australia, New Zealand, Spain, Argentina	No	Faster and more controllable than natural freezing; reduces field exposure risk; cannot fully replicate natural aroma profile. In an experimental comparison on Muscat of Alexandria, liquid nitrogen freezing produced greater increases in terpenes, esters and aldehydes than ultra-rapid mechanical freezing, with the highest sensory scores among artificial methods, but both remained chemically distinct from naturally frozen ice wine
Supraextraction	Post-harvest freezing, berries fully thawed before pressing	Same warmer-climate contexts as cryoextraction	No	Produces fresher, better-structured wines vs. traditional maceration, but remains distinct from natural ice wine
Fractional cryoconcentration	Partial freezing of pressed juice or must (not whole berries), followed by physical separation of the concentrated unfrozen fraction from the ice fraction	Used to raise sugar/must concentration where natural or whole-berry freezing is impractical	No	Concentrates must composition directly rather than via berry tissue disruption; distinct in mechanism from both cryoextraction and supraextraction, which act on whole berries before pressing

## Data Availability

No new data were created or analysed in this study. Data sharing is not applicable to this article.

## Abbreviations

The following abbreviations are used in this manuscript:

AR(1)	Autoregressive Model of Order 1
Brix	Degrees Brix (sugar content scale)
C6, C9, C13	Carbon chain length (volatile organic compounds)
CMIP6	Coupled Model Intercomparison Project Phase 6
DJF	December–January–February (winter season index)
E-nose	Electronic nose (artificial sensory system)
ETCCDI	Expert Team on Climate Change Detection and Indices
GC-MS	Gas Chromatography–Mass Spectrometry
HS-SPME	Headspace Solid-Phase Microextraction
HS-SPME-GC-MS	Headspace Solid-Phase Microextraction–Gas Chromatography–Mass Spectrometry
IPCC	Intergovernmental Panel on Climate Change
LN	Liquid Nitrogen (freezing method)
VOC	Volatile Organic Compounds
MIR	Mid-Infrared Spectroscopy
NIR	Near-Infrared Spectroscopy
NOAA	National Oceanic and Atmospheric Administration
OAV	Odour Activity Value
OIV	International Organisation of Vine and Wine
PLS-DA	Partial Least Squares Discriminant Analysis
SAT	Sum of Active Temperatures
SSP	Shared Socioeconomic Pathway
SSP1-2.6	Low-emissions scenario (CMIP6)
SSP5-8.5	High-emissions scenario (CMIP6)
TDN	1,1,6-Trimethyl-1,2-dihydronaphthalene
TN10p	Cold nights index (days below 10th percentile of Tmin)
TN90p	Warm nights index (days above 90th percentile of Tmin)
TNn	Coldest night (annual minimum of daily minimum temperature)
TNx	Warmest night (annual maximum of daily minimum temperature)
ToE	Time of Emergence
TX10p	Cold days index (days below 10th percentile of Tmax)
TX90p	Warm days index (days above 90th percentile of Tmax)
TXn	Coldest day (annual minimum of daily maximum temperature)
UF	Ultra-Fast Mechanical Freezing
